# Interpreting the Medical Termination of Pregnancy Act by primary care providers in rural Karnataka: implications on safe abortion services

**DOI:** 10.1186/1753-6561-6-S5-P14

**Published:** 2012-09-28

**Authors:** Maya Annie Elias, Arima Mishra, HY Vijayashree, Mamata R Patil, MH Anil, MR Raveesha, N Devadasan, Patrick Van Dessel

**Affiliations:** 1 Institute of Public Health, Bangalore, India; 2 Institute of Tropical Medicine, Antwerp, Belgium

## Introduction

Evidence shows that eight per cent of maternal mortality in India is caused by unsafe abortions. India was one of the pioneer countries to legalize its abortion services as early as 1971. The Medical Termination of Pregnancy Act (MTP) is an enabling regulation, which allows MTP on both medical and social grounds and thus facilitates access to safe abortion services. However, despite this supposedly progressive law, which has existed for more than four decades, ‘illegal’ and unsafe abortions are still a reality in India particularly in rural areas. There is a large body of research on MTP that examines barriers to access safe abortion services from the users’ perspectives. This paper turns the research gaze from the users to the providers particularly at the primary health care level to examine the persistence of illegal and unsafe abortions in rural Karnataka.

## Methods

We conducted in-depth interviews with primary care providers that include medical officers in primary health centres and focus group discussions with frontline health workers including Auxiliary Nurse Midwives (ANMs), Lady Health Visitors (LHVs) and Accredited Social Health Activists (ASHAs). We collected data in two districts in Karnataka from July to December 2011. Data were collected as part of a larger research project ‘Health System Stewardship and Regulations in Vietnam, India and China’ (HESVIC), which looked at how regulations and through it governance, affect equitable access to quality maternal health care.

## Results

Results of the study showed that (a) very few of the medical officers were trained in MTP though many of them offered the services; (b) there was a perceived disjunction between clinical and legal training; MTP training was perceived as a mere ‘legal cover’ and not necessarily equipping providers with additional skills to provide MTP services; (c) in the absence of legal training, providers justify the provision of delivery of service through a range of explanations including “being ethical catering to patients’ demands”, “we provide abortion services not MTP”, “abortions through oral pills are out-patient cases and hence are not reported”; (d) MTP was largely perceived as a ‘restrictive regulation’ than enabling. Such perception was influenced by several factors like the mandatory legal training, exclusion of non-allopathic primary providers, inadequate dissemination of the regulations including amendments, lack of sensitization training of providers and health workers and providers’ own cultural beliefs (those who subscribe to a pro-life approach); and, (e) boundaries between legal and illegal were often drawn at ‘married/unmarried’ (the latter as illegal), ‘less than and more than 12 weeks of pregnancy’. Such boundaries were shaped by a cultural and a skewed reading of the legislation.

## Discussion

Apart from indicating towards poor monitoring of a regulation, the analysis shows how the providers’ interpretation of the MTP Act and training status results in provision of abortion services that are ‘illegal’ and potentially unsafe. The findings of the study indicate an urgent need for sensitization training for frontline health workers and paramedical staff on MTP regulation, more accountability from district authorities in facilitating training of primary health care providers and periodic refresher training for the trained doctors to sensitize them on newer technology and also on amendments of the regulation.

## Competing interests

None declared.

## Funding statement

The study was funded by the European Commission funded HESVIC project (2009-2012).

